# “Clock dial pattern”, a radiologic clue to neuro-chikungunya diagnosis: a case series

**DOI:** 10.1055/s-0044-1779033

**Published:** 2024-01-29

**Authors:** Pedro Henrique Almeida Fraiman, Mariana Freire, Bruno Fernandes, Felipe Palitot, Nathalia Mota, Eduardo Sequerra, Glauco Santos, Mario Emilio Dourado, Clecio de Oliveira Godeiro-Junior, Manuel Moreira-Neto

**Affiliations:** 1 Universidade Federal do Rio Grande do Norte, Hospital Universitário Onofre Lopes, Divisão de Neurologia, Natal RN, Brazil.; 2 Universidade Federal de São Paulo, Departamento de Neurologia e Neurocirurgia, São Paulo SP, Brazil.; 3 Universidade Federal do Rio Grande do Norte, Hospital Universitário Onofre Lopes, Divisão de Radiologia, Natal RN, Brazil.; 4 Universidade Federal do Rio Grande do Norte, Instituto do Cérebro, Natal RN, Brazil.; 5 Hospital Giselda Trigueiro, Divisão de Infectologia, Natal RN, Brazil.

**Keywords:** Chikungunya Virus, Myelitis, Arbovirus Infections, Vírus Chikungunya, Mielite, Infecções por Arbovírus

## Abstract

**Background** Chikungunya is a mosquito-borne disease caused by the chikungunya virus (CHIKV) and can lead to neurological complications in severe cases.

**Objective** This study examined neuroimaging patterns in chikungunya cases during two outbreaks in Brazil to identify specific patterns for diagnosis and treatment of neuro-chikungunya.

**Methods** Eight patients with confirmed chikungunya and neurological involvement were included. Clinical examinations and MRI scans were performed, and findings were analyzed by neuroradiologists. Data on age, sex, neurological symptoms, diagnostic tests, MRI findings, and clinical outcomes were recorded.

**Results** Patients showed different neuroimaging patterns. Six patients exhibited a “clock dial pattern” with hyperintense dotted lesions in the spinal cord periphery. One patient had thickening and enhancement of anterior nerve roots. Brain MRI revealed multiple hyperintense lesions in the white matter, particularly in the medulla oblongata, in six patients. One patient had a normal brain MRI.

**Conclusion** The “clock dial pattern” observed in spinal cord MRI may be indicative of chikungunya-related nervous system lesions. Isolated involvement of spinal cord white matter in chikungunya can help differentiate it from other viral infections. Additionally, distinct brainstem involvement in chikungunya-associated encephalitis, particularly in the rostral region, sets it apart from other arboviral infections. Recognizing these neuroimaging patterns can contribute to early diagnosis and appropriate management of neuro-chikungunya.

## Resumo

**Antecedentes** A chikungunya é uma doença transmitida por mosquitos causada pelo vírus chikungunya (CHIKV) e pode levar a complicações neurológicas em casos graves.

**Objetivo** Este estudo examinou padrões de neuroimagem em casos de chikungunya durante dois surtos no Brasil para identificar padrões específicos para o diagnóstico e tratamento de neurochikungunya.

**Métodos** Oito pacientes com chikungunya confirmada e envolvimento neurológico foram incluídos. Exames clínicos e ressonâncias magnéticas (RM) foram realizados, e os achados foram analisados por neurorradiologistas. Dados sobre idade, sexo, sintomas neurológicos, testes diagnósticos, achados de RM e desfechos clínicos foram registrados.

**Resultados** Os pacientes apresentaram diferentes padrões de neuroimagem. Seis pacientes apresentaram um “padrão de mostrador de relógio” com lesões pontilhadas hiperintensas na periferia da medula espinhal. Um paciente apresentou espessamento e realce das raízes nervosas anteriores. A RM do cérebro revelou múltiplas lesões hiperintensas na substância branca, especialmente no bulbo em seis pacientes. Um paciente apresentou uma RM cerebral normal.

**Conclusão** O “padrão de mostrador de relógio” observado na RM da medula espinhal pode ser indicativo de lesões do sistema nervoso relacionadas à chikungunya. O envolvimento isolado da substância branca da medula espinhal na chikungunya pode ajudar a diferenciá-la de outras infecções virais. Além disso, o envolvimento distinto do tronco cerebral na encefalite associada à chikungunya, especialmente na região rostral, a distingue de outras infecções por arbovírus. O reconhecimento desses padrões de neuroimagem pode contribuir para o diagnóstico precoce e manejo adequado da neurochikungunya.

## INTRODUCTION

Chikungunya is a mosquito-borne disease caused by the chikungunya virus (CHIKV). Among epidemic outbreaks of CHIKV, rare neurological manifestations were described in some severe patients. 1 Neuroimaging is frequently normal in most patients, but abnormalities could be seen especially in those diagnosed with encephalitis and myelitis. 1

Our study presents a case series of neuroimaging patterns during two epidemic outbreaks in 2016 and 2019 in the state of Rio Grande do Norte, Brazil. These patients developed new and more specific patterns. The recognition of these patterns might help to narrow differential diagnosis in neural-infectious diseases and guide early diagnosis and treatment of neuro-chikungunya.

## METHODS

We conducted a case study and included 8 patients from one center, admitted to a tertiary care hospital during two epidemics of chikungunya outbreaks in 2016 and 2019. All subjects had confirmed diagnosis of chikungunya through serological or molecular tests (performed by the Rio Grande do Norte state secretariat of public health, Table 1 ), had fulfilled 2015 CDC criteria of neuroinvasive infection by arboviruses 2 and underwent clinical examination by a neurologist. Patients were not included if had vaccination one month before the onset of symptoms; if presented a previous diagnosis of neurologic diseases; or if they had a concomitant infection with dengue or Zika virus. Patients had MRI studies performed in 1.5 Tesla scanners (GE HDxt and Siemens MAGNETOM Avanto) using sagittal and axial images of cervical, thoracic, and/or lumbar spine in T1WI, T2WI, and STIR sequences, with and without paramagnetic contrast agent (gadolinium). Additionally, six patients underwent brain MRI studies when symptoms suggested encephalitis. Images were analyzed by an experienced neuroradiology team.

**Table 1:** Patients and their neuroimaging characteristics.

Patient	Age	Sex	Year of occurrence	Neurological presentation	Discharge clinical outcome	Diagnostic test	Time from beginning of symptoms (weeks)	Spinal cord MRI	Brainstem MRI	Brain MRI
1	36	Female	2016	Acute respiratory failure, flaccid and areflexic tetraparesis, seizures, Babinski sign, and bladder disfunction	Sensory deficit and neuropathic pain.	IgM + , serum	4	T2 hyperintense small dotted lesions, mainly on the periphery of the spinal cord	T2 hyperintense lesions in the medulla	T2 hyperintense lesions in deep white matter; no restricted diffusion on DWI
2	75	Female	2016	Lower limbs pain, acute respiratory failure, difficulty walking, and bladder disfunction	Axonal lumbosacral polyradiculoneuropathy	IgM + , serum	4	T2 hyperintense small dotted lesions, mainly on the periphery of the spinal cord	Absence of findings	T2 hyperintense lesions in white matter mainly in the periventricular region, non-specific, may correspond to microangiopathy
3	70	Female	2016	Acute flaccid paraplegia, urinary retention, and constipation	Hypoesthesia under T8 level	IgM + , serum	2	T2 hyperintense and Gd-enhancing small dotted lesions, mainly on the periphery of the spinal cord (cervical and thoracic spine).	Discrete T2 hyperintense focal lesions	Absence of findings
4	49	Male	2016	Lumbar pain and flaccid areflexic asymmetric paraparesis and bladder disfunction	Neurogenic bladder	RT-PCR, serum	4	Thickening and enhancement of the anterior nerve roots of the cauda equina	No data available	No data available
5	58	Male	2019	Superficial and deep hypoesthesia with a sensitive level in T10	Clinical recovery	IgM + , serum	4	T2 hyperintense small dotted lesions in multiple segments, mainly on the periphery of the spinal cord	No data available	No data available
6	75	Female	2016	Quadriparesis (lower extremity predominance), acute flaccid paraparesis, thoracic sensory level, bladder disfunction	Clinical recovery	IgM + , serum	4	Absence of findings	T2 hyperintense dotted lesions, mainly on the anterior region of the medulla	Multifocal hyperintense T2 lesions spread through the cerebral white matter
7	60	Male	2019	Acute respiratory failure, paresis of the lower extremity and, bladder disfunction	Encephalomyeloradiculitis	IgM + , serum	6	T2 hyperintense small dotted lesions in multiple segments, mainly on the periphery of the spinal cord	T2 hyperintense small lesions in the anterior medulla. No enhancement detected	Small multifocal T2 hyperintense lesions spread in cerebral white matter with the confluence in the sublenticular region, no enhancement detected, and with restricted diffusion on DWI.
8	58	Male	2019	Bladder dysfunction, constipation, flaccid quadriparesis	Clinical improvement with corticosteroids pulse therapy	RT-PCR, serum	1	T2 hyperintense small dotted lesions in multiple segments, mainly on the periphery of the spinal cord	Absence of findings	Absence of findings

Clinical measures were recorded by interrogation and relevant information was obtained in the medical records, including age, sex, neurological presentation, diagnostic test, MRI findings, and clinical outcome at discharge.

This case study was approved by the local ethics committee (Ethics Committee in Research, Universidade Federal do Rio Grande do Norte, 09144819.1.0000.5292). Free and informed consent was given by all patients.

## RESULTS

We included 8 patients (50%, male), with a mean age of 59 years old (standard deviation ± 13,9 years; range: 36 to 75-year-old), a median time at imaging exam from the beginning of symptoms of 4 weeks (range: 1 to 6 weeks). Their clinical and laboratory data are shown in Table 1 .

**Figure 1:**
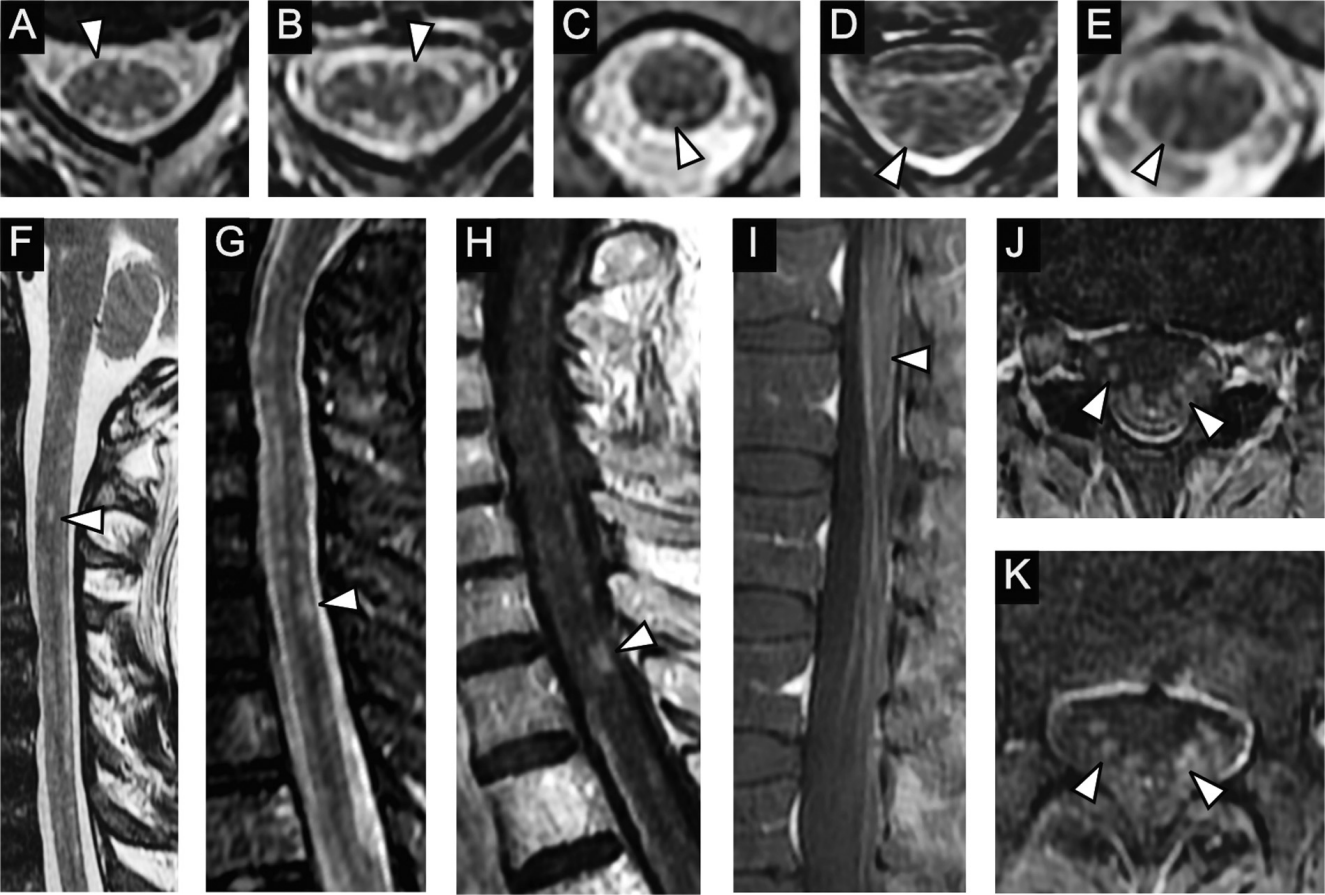
Spinal cord magnetic MRI features of patients with CHIKV-associated encephalomyelitis. (A, B) patient 7 - Axial FSE T2WI images demonstrating small dotted lesions (arrowheads) with high signal in the periphery of the spinal cord (the “ *clock dial pattern* ”). (C-E) patient 1 - Axial T2-WI images showing multiple small dotted or linear lesions (arrowheads) with high signal in the periphery of the cervical and thoracic spinal cord (features of the “clock *dial pattern* ”). (F) patient 7 - Sagittal FSE T2WI image demonstrating small dotted lesions (arrowhead) with high signal in the periphery of the spinal cord (features of the “clock *dial pattern* ”). (G, H) patient 3 - Sagittal STIR and FSE post-contrast T1WI images of the cervical spine, demonstrating small multiple T2 high signal lesions with post-contrast enhancement in the periphery of the spinal cord (arrowhead). Similar features are seen in the thoracic spinal cord of this patient (not shown). (I, J, K) patient 4 - Sagittal and axial post-contrast T1WI images, demonstrating thickening and post-contrast enhancement (arrowheads) of the anterior cauda equina roots, a pattern usually seen in Guillain-Barré Syndrome.

In six cases (patients 1, 2, 3, 5, 7, and 8), spinal cord MRI presented a singular pattern of T2WI hyperintense small dotted lesions in multiple segments, mainly on the periphery of the spinal cord ( Figure 1 ). There is no mass effect or enhancement by paramagnetic contrast media in most cases – in patient 3, however, there was enhancement after Gadolinium mainly in the cervical ( Figure 1 G, H ) and thoracic lesions. This pattern of multiple peripheral lesions resembles a “clock dial” configuration when observed in axial sections. One of the patients (patient 4) has shown thickening and enhancement by the contrast in the anterior nerve roots of the cauda equina ( Figure 1 I-K ).

**Figure 2:**
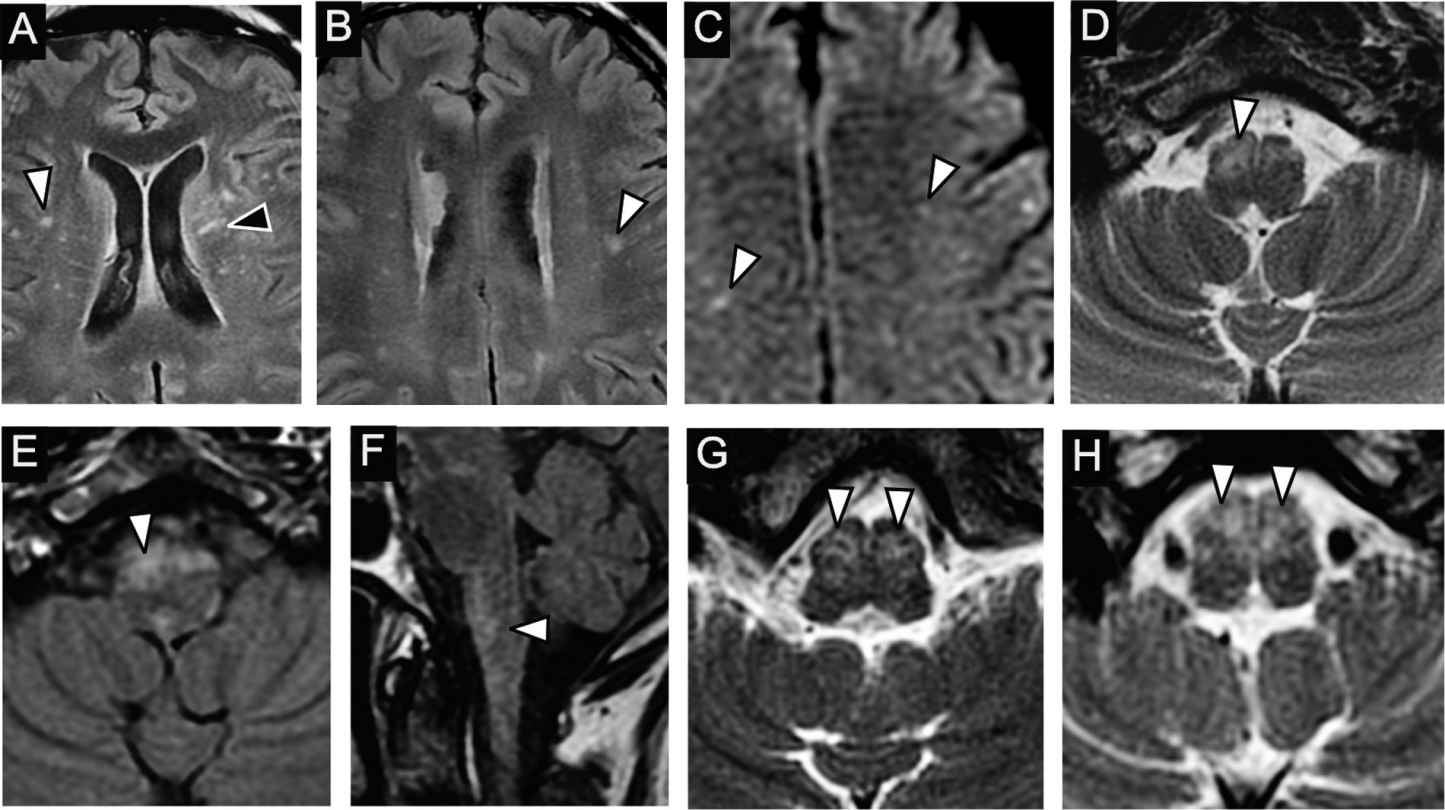
Brain MRI features of patients with CHIKV-associated encephalomyelitis. (A-F) patient 7 - (A, B) Axial T2WI FLAIR images showing multiple small and scattered punctiform (white arrowhead) or linear lesions (black arrowhead), with high signal in T2WI, no mass effect, enhancement, or restriction on diffusion sequence in the white matter of both brain hemispheres. Axial DWI images (C) revealed restriction to the diffusion of water in some of these lesions (arrowheads). Axial T2WI (D), Axial (E) and Sagittal (F) FLAIR images showing small lesions with high signal (arrowheads), no mass effect or enhancement, in the anterior regions of the medulla oblongata. (G, H) patient 6 - Axial images in FSE T2WI (G, H) showing small dotted or linear lesions (arrowheads) with high signal in T2WI, no mass effect, enhancement, or restriction on diffusion sequence, in the medulla oblongata.

Six patients underwent brain MRI ( Figure 2 ). Three cases (patients 1, 6, and 7) have shown T2WI hyperintense small multiple spotlights and randomly distributed lesions in supratentorial white matter and brainstem, especially in the anterior medulla ( Figure 2 D-F ). The lesions in patient 7 have shown abnormal restricted diffusion in DWI sequence ( Figure 2 C ). One of the brain MRIs was normal (patient 8). Their imaging findings are in Table 1 .

## DISCUSSION

Herein, we describe a case series of Neuro-Chikungunya during two epidemic outbreaks in 2016 and 2019. We suggest that T2 hypertense small peripheral white matter dotted or linear lesions, with or without contrast enhancement, in the spinal cord MRI, the here-named “clock dial pattern,” could be a radiological clue to distinguish Chikungunya virus-associated nervous system lesions from those caused by other neurotropic arboviruses.

CHIKV is an arbovirus of the *Togaviridae* family that is recognized for its neurovirulence and neurological complications. 3 These neurological complications are not specific to CHIKV infection among arboviruses, they are also reported in dengue and zika virus infections, other arboviruses that cocirculate with and share the same vector of CHIKV.

Among patients that show neurological complications in CHIKV infection, neuroimaging abnormalities are variable. The abnormalities in the spinal cord range from findings suggestive of demyelinating pathology to hyperintensity in FLAIR sequences, suggestive of extensive longitudinal myelitis. 1 There is a case report, during the Brazilian epidemic, of predilection for the anterior horn of the spinal cord in a case of chikungunya and dengue coinfection, associated with acute transverse myelitis. 4 The neuroimaging findings of patients with clinical signs of encephalitis or encephalopathy are also unspecific and variable. There are descriptions of edema or non-specific hemorrhage on brain computed tomography; there are also hyperintensities in T2WI and FLAIR and abnormal restricted diffusion on DWI in several areas on brain MRI, but many cases do not show any abnormalities. 1 5 6 7

The patients in our series who had neurological manifestations compatible with myelitis displayed the “clock dial pattern.” In most patients, the “clock dial pattern” is observed without enhancement after contrast (only one of the patients displayed lesions enhancement after contrast). These peripheral lesions in the white matter could be associated with direct virus injury through retrograde dissemination due to nerve root involvement. This pattern of lesions resembles “clock dials” due to the peripheral distribution of lesions in the white matter of the spinal cord when seen in axial sections ( Figure 1 A-I ). Among other viral infections, dengue myelitis shows a pattern of gray matter preferential involvement, mainly in the anterior horn of the spinal cord, including cases of longitudinally extensive transverse myelitis. 8 9 This pattern of anterior horn involvement is also seen in enterovirus infections. 8 Therefore, the particular involvement of the spinal cord white matter might be a helpful feature suggestive of differential diagnosis of neuro-chikungunya from other viral infections in the central nervous system.

Other findings also found in the spinal cord MRIs were the thickening and enhancement by the contrast of the anterior nerve roots of the cauda equina ( Figure 1, J-K ). This patient has a clinical presentation compatible with Guillain-Barré Syndrome, which explains this different morphological pattern of the nerve roots and peripheral nerves. 10 This pattern is not particular to CHIKV infections, it is also seen in dengue, zika, and other viruses related to Guillain-Barré Syndrome manifestations. 8 10

Among patients with clinical signs of encephalitis, brain MRI has shown multiple and scattered white matter hyperintense lesions on T2WI sequences, without enhancement after contrast or mass effect. Brainstem involvement has presented a predilection for the medulla oblongata region, regarding a similar pattern of multiple dotted and linear hyperintensities on T2WI sequences, as seen in the spinal cord, mainly in the rostral region, particularly in medullary pyramids ( Figure 2, A-H ). In dengue virus-associated encephalitis, the main differential diagnosis of CHIKV-associated encephalitis, brainstem involvement is not common and usually spares the rostral region, preferentially affecting the substantia nigra of the midbrain. 8 We need to consider that the overlapping between gliosis related to microangiopathy and CHIKV-related lesions is a limitation in elderly patients. In young patients, however, these findings must be considered as secondary to CHIKV.

Limitations of this study include that it is retrospective and thus dependent on the accuracy of existing medical records. We were limited by the observational nature of the study, including analysis of patients at different times of their disease course, as well as missing data. These limitations are inherent to the study design. The number of cases is small, and a larger number of patients is needed to better estimate the frequency of the occurrence of the “clock dial pattern” in neuro-chikungunya cases. Nevertheless, the strength of our study involves describing an imaging pattern reproducible in two different epidemic settings ( Table 1 ), which helps to narrow the differential diagnosis of a rare but severe complication of a common disease, thus possibly decreasing morbidity and mortality and improving the outcome of these patients.

In general, neurological complications are rare in chikungunya but have high morbidity and mortality. Additionally, it is possible that the “clock dial pattern” is an early manifestation of the disease. Then, clinicians, neurologists, and radiologists should be aware of and recognize this pattern to support an early correct diagnosis.
